# Preparation of Antioxidant Peptides from Salmon Byproducts with Bacterial Extracellular Proteases

**DOI:** 10.3390/md15010004

**Published:** 2017-01-11

**Authors:** Ribang Wu, Leilei Chen, Dan Liu, Jiafeng Huang, Jiang Zhang, Xiao Xiao, Ming Lei, Yuelin Chen, Hailun He

**Affiliations:** 1 School of Life Science, State Key Laboratory of Medical Genetics, Central South University, Changsha 410013, China; ribang.wu@gmail.com (R.W.); liudan.forever@163.com (D.L.); 1608110217@csu.edu.cn (J.H.); zhangjiang915@163.com (J.Z.); 162511013@csu.edu.cn (X.X.); leiming@csu.edu.cn (M.L.); lumc96@hotmail.com (Y.C.); 2 Institute of Agro-Food Science and Technology & Shandong Provincial Key Laboratory of Agro-Products Processing Technology, Shandong Academy of Agricultural Sciences, Jinan 250100, China; chenleilei8210@163.com

**Keywords:** bacterial extracellular proteases, antioxidant peptide, enzymatic hydrolysis, peptide purification, evaluation of antioxidant activity

## Abstract

Bacterial extracellular proteases from six strains of marine bacteria and seven strains of terrestrial bacteria were prepared through fermentation. Proteases were analyzed through substrate immersing zymography and used to hydrolyze the collagen and muscle proteins from a salmon skin byproduct, respectively. Collagen could be degraded much more easily than muscle protein, but it commonly showed weaker antioxidant capability. The hydrolysate of muscle proteins was prepared with crude enzymes from *Pseudoalteromonas* sp. SQN1 displayed the strongest activity of antioxidant in DPPH and hydroxyl radical scavenging assays (74.06% ± 1.14% and 69.71% ± 1.97%), but did not perform well in Fe^2+^ chelating assay. The antioxidant fractions were purified through ultrafiltration, cation exchange chromatography, and size exclusion chromatography gradually, and the final purified fraction U2-S2-I displayed strong activity of antioxidant in DPPH, hydroxyl radical scavenging assays (IC_50_ = 0.263 ± 0.018 mg/mL and 0.512 ± 0.055 mg/mL), and oxygen radical absorption capability assay (1.960 ± 0.381 mmol·TE/g). The final purified fraction U2-S2-I possessed the capability to protect plasmid DNA against the damage of hydroxyl radical and its effect was similar to that of the original hydrolysis product. It indicated that U2-S2-I might be the major active fraction of the hydrolysate. This study proved that bacterial extracellular proteases could be utilized in hydrolysis of a salmon byproduct. Compared with collagen, muscle proteins was an ideal material used as an enzymatic substrate to prepare antioxidant peptides.

## 1. Introduction

Oxidative stress is an imbalance between oxidation and antioxidation, caused by external or internal stimulation. Reactive oxygen species (ROS), such as superoxide anion radicals (O_2_^●−^), hydrogen peroxide (H_2_O_2_), hydroxyl radicals (^●^OH), and peroxyl radicals (ROO^●^), can damage DNA, proteins, and membrane systems, which is the significant nosogenesis of chronic diseases [1], including cancer [2], heart disease [3], and Alzheimer’s [4]. In addition, free-radical-mediated lipid peroxidation can lead to food spoilage and the generation of potentially toxic products. Although an endogenous antioxidant system containing antioxidant enzymes (superoxide dismutase, glutathione S-transferase, and catalase) and antioxidant substrates (glutathine and victamin C) can scavenge ROS to protect cells in vivo, the excessive ROS that cannot be removed promptly still damage cells and tissues. In the food industry, synthetic antioxidants, such as butylated-hydroxytoluene (BHT), butylated-hydroxyanisole (BHA), tertbutyl-hydroquinone (TBHQ), and propyl gallate (PG), have been used during food processing, but existing potentially toxic effect on health. Antioxidant peptides are a series of oligopeptides with specific amino acid sequences, which can scavenge free radicals or inhibit the generation of ROS.

A lot of studies have reported that the enzymatic hydrolysis of protein is an effective way to prepare novel bioactive peptides, including antioxidant peptides. Numerous protein resources were found to be ideal materials of antioxidant peptides preparation, such as blacktip shark skin [5], blue mussel protein [6], cod protein [7], skate skin [8], oysters [9], and so on. Meanwhile, during fish processing, a great amount of fish byproducts, such as fish skin, bone frame, and fins, were processed into animal feed, which were not utilized comprehensively and therefore caused a severe waste of protein resources. Recent studies have reported that byproducts with abundant protein can be recycled and used to prepare bioactive peptides. Series of peptides can be released from the parental protein by enzymatic hydrolysis, and they may possess different kinds of biological activities, such as antioxidant, inhibition of angiotensin I-converting enzyme (ACE), antibiotic, anti-freezing, and so on [5,10]. Salmon is a valuable and popular edible fish worldwide [11]. Previous studies have reported that salmon byproducts could be hydrolyzed by various proteases to prepare bioactive peptides. Sathiel et al. extracted functional and nutritional peptides from salmon head hydrolysates with different enzymes [12]. Ahn et al. used six kinds of proteases to hydrolyze protein from the salmon pectoral fin, and found that peptic hydrolysates exhibited antioxidant and anti-inflammatory activities [13]. Most of the enzymes used in these researches are commercial proteases, such as pepsin, trypsin, chymotrypsin, papain, flavourzyme, and so on. However, non-commercial proteases were seldom reported in peptide preparation.

Recently, marine bacteria have become important sources for the selection of novel enzymes. The proteases secreted by marine bacteria play an important role in the decomposition of organic nitrogen in oceans, and therefore they have incomparable advantages in hydrolyzing marine-sourced protein [14]. Compared with land proteases, marine proteases usually have higher catalytic efficiency in hydrolyzing marine protein, such as fish skin, muscle, and bone frame [15,16]. The cleavage site of proteases from different bacteria also varies widely. With these enzymes, peptides obtained from protein hydrolysate may possess different amino acid sequences. It would be beneficial to discover more new bioactive peptides. This study aimed at investigating the effect of several extracellular proteases from bacteria on hydrolyzing the protein of salmon byproduct. The antioxidant activity of each hydrolysate and purified fraction was evaluated in order to verify the possibility of application.

## 2. Results and Discussion

### 2.1. Substrate-Immersing Zymography of Bacterial Extracellular Proteases

**Figure 1:**
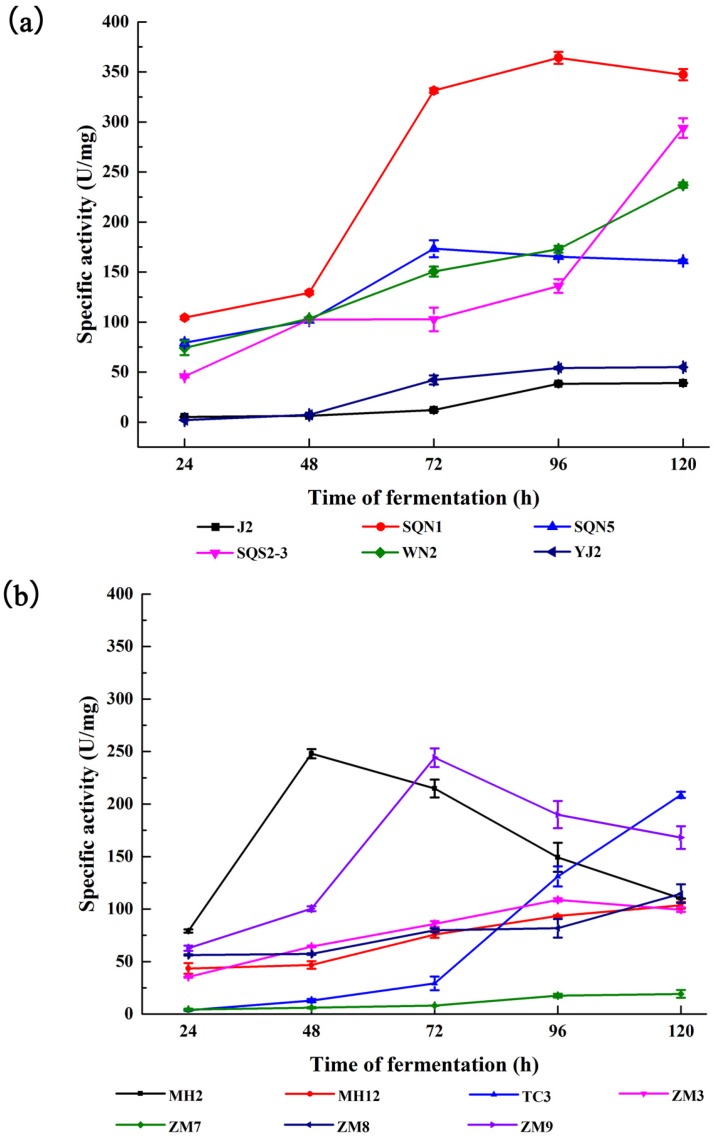
Time course specific activity of bacterial crude enzymes came from marine bacteria (**a**) and fresh water bacteria (**b**) during five-day fermentation. Values are displayed as means ± SD (*n* = 3).

The bacterial fermentation products were obtained every 24 h for five days. Then the enzymatic activity and concentration of total protein were quantified with the Folin’s phenol and BCA method to calculate specific activity and the time course of protease activity, respectively (Figure 1). It was obvious that *Pseualtermonas* sp. SQN1 produced a protease with much higher specific activity (347.27 ± 5.58 U/mg) compared with other tested bacteria from sea water. The enzymatic activity could become stable after 72 h fermentation, which was faster than *Vibrio* sp. SQS2-3 and *Vibrio* sp. SWN2. Bacteria from fresh water commonly display lower specific activity, such as *Bacillus* sp. MH12, *Aeromonas* sp. ZM3, and *Aeromonas* sp. ZM7. In addition, *Exiguobacterium* sp. MH2 and *Pseudomonas* sp. ZM9 could produce proteases in a shorter time, but the specific activity decreased rapidly after two and three days, respectively.

**Figure 2:**
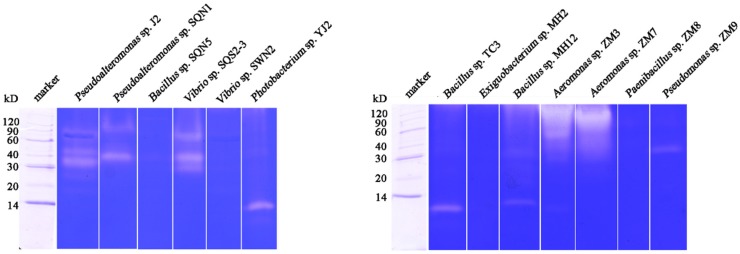
Substrate immersing zymography of different bacterial extracellular proteases.

The enzymatic activity and protease composition of each bacterial fermentation products after five days were detected through substrate immersing zymography. The result of zymography was summarized in Table 1, including the amount of proteases, the total protein concentration of crude enzyme, specific activity, and the molecular weight of each active band. As shown in Figure 2, crude enzymes from *Pseudoalteromonas* sp. J2, *Pseudoalteromonas* sp. SQN1, *Vibrio* sp. SQS2-3, and *Bacillus* sp. MH12 contained several proteases. Only one kind of protease was displayed in the lanes of *Photobacterium* sp. YJ2, *Bacillus* sp. TC3, and *Pseudomonas* sp. ZM9. The enzymes from *Aeromonas* sp. ZM3 and *Aeromona* sp. ZM7 showed the strongest enzymatic activity on casein. The crude enzyme from *Bacillus* sp. SQN5, *Vibrio* sp. SWN2, *Exiguobacterium* sp. MH2, and *Paenibacillus* sp. ZM8 did not display the ability of degrading casein in zymography. Theoretically, multi-proteases possess more enzymatic cleavage sites, which could hydrolyze protein more effectively. The antioxidant peptides were commonly separated from the hydrolysates with a relatively higher hydrolysis degree, so multi-proteases might be more suitable for antioxidant peptide preparation. Wang et al. reported that hydrolyzing collagen from croceine croaker with pepsin and trypsin could obtain peptides with higher antioxidant activity compared with preparation with pepsin or trypsin alone [17]. Meanwhile, other factors should be considered when choosing enzymes, such as enzymatic activity and substrate binding capability. Higher enzymatic activity and substrate binding capability could shorten the reaction time and release antioxidant peptides more easily. On the other hand, some crude proteases contain a lot of extracellular polysaccharide, which makes the samples sticky and impedes the migration of proteases in gel, just like the enzymes from *Aeromonas* sp. ZM3 and *Aeromonas* sp. ZM7. The byproducts of fermentation would increase cost and difficulty in purification, so these two enzymes were not selected. Zymography analysis of bacterial proteases was necessary and helpful to choose enzymes by screening before the preparation of antioxidant peptides. With zymography analysis, the enzymatic activity, the amount of proteases, purification difficulty, or even the type of each protease could be forecasted to a certain degree.

### 2.2. Hydrolysis of Salmon Protein

**Figure 3:**
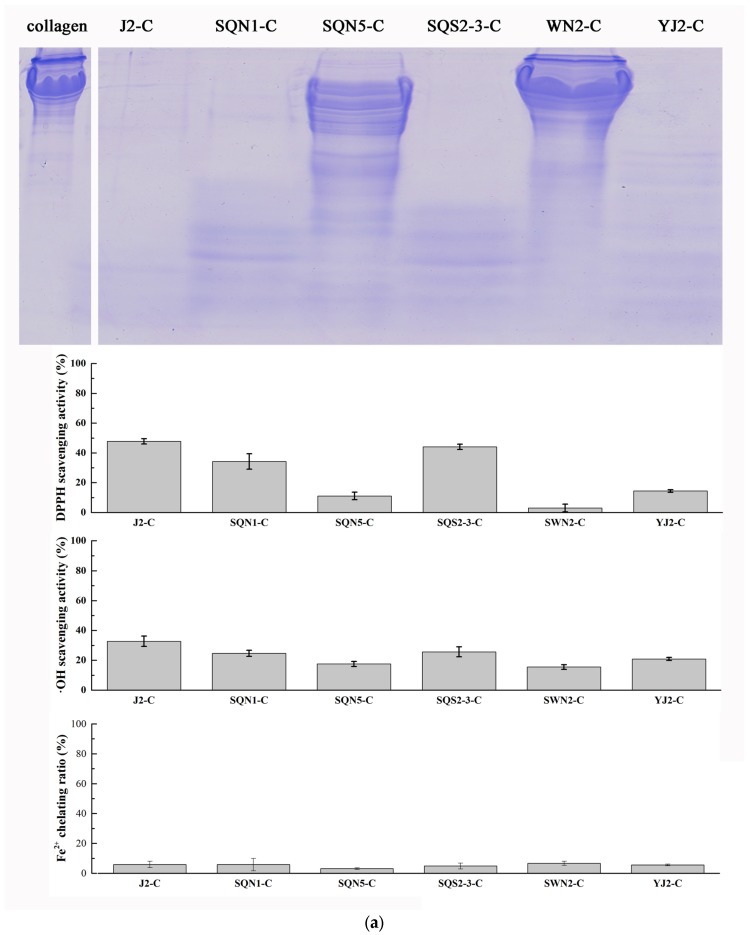

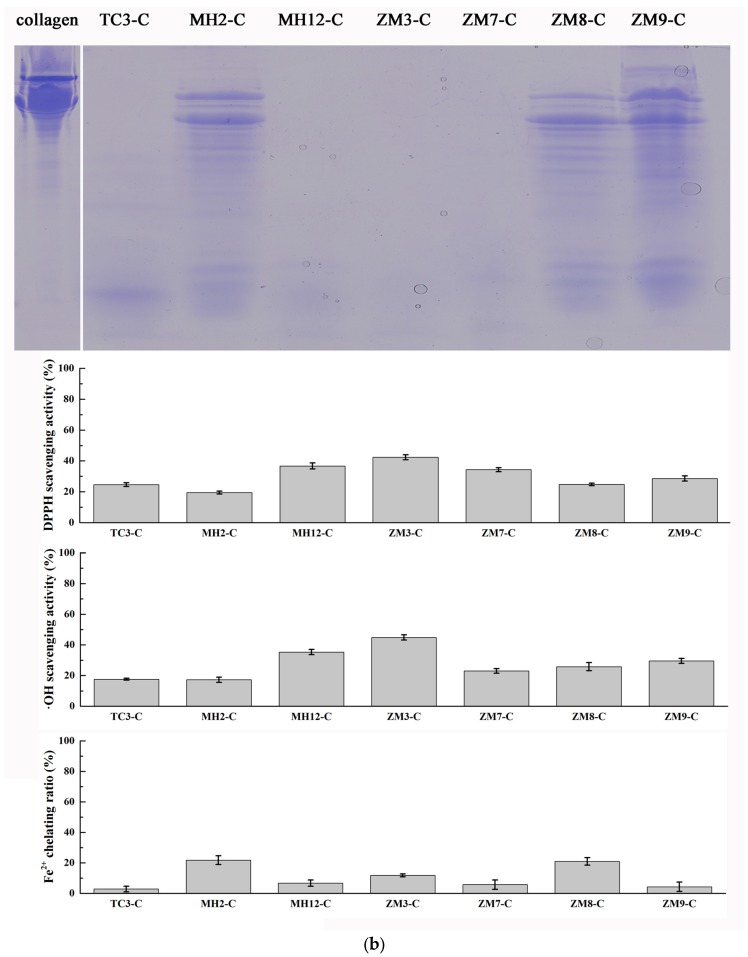

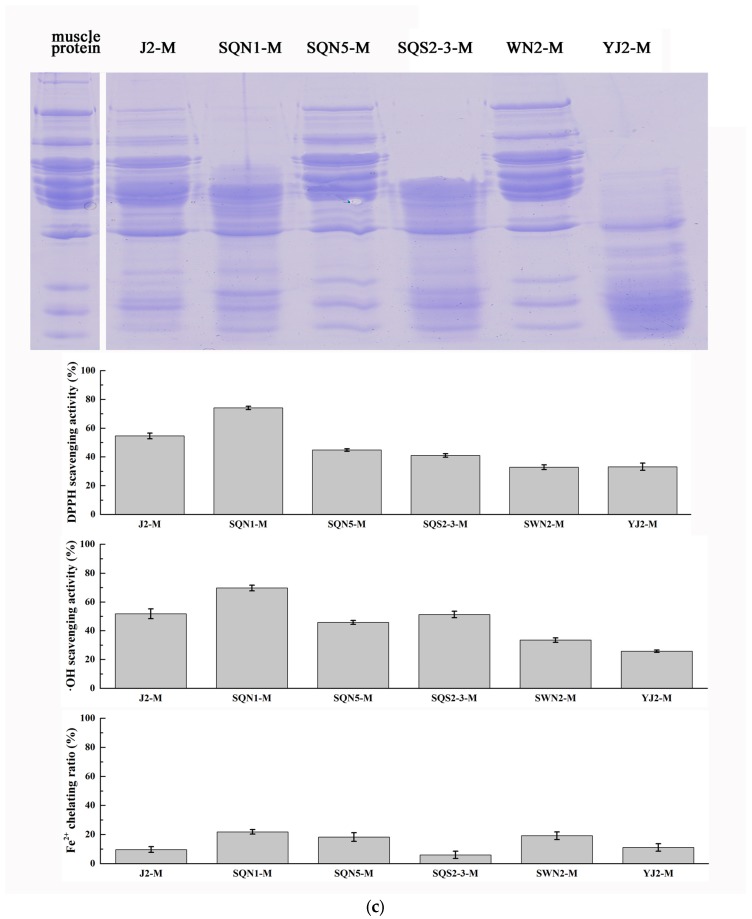

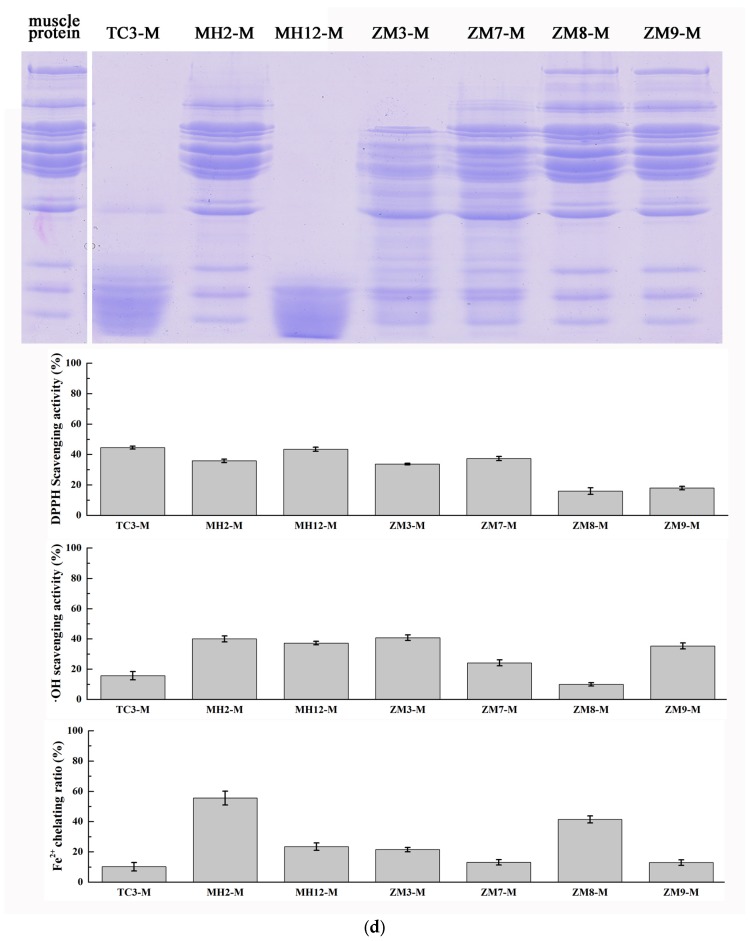
Hydrolysis results of (**a**) salmon collagen with sea water bacterial proteases; (**b**) salmon collagen with fresh water bacterial proteases; (**c**) salmon muscle protein with sea water bacterial proteases; and (**d**) salmon muscle proteins with fresh water bacterial proteases in SDS-PAGE, and the antioxidant activity measured with DPPH, hydroxyl radical scavenging assays, and ferrous ion chelating assay (*n* = 3).

**Figure 4:**
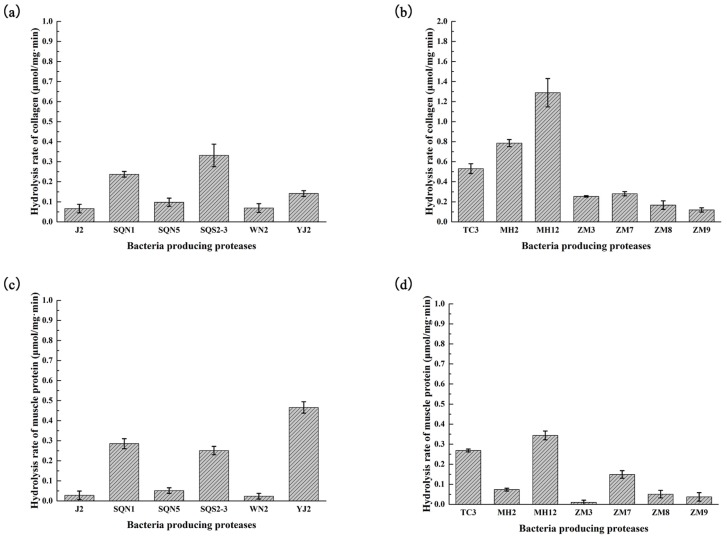
Hydrolysis rate of (**a**) marine bacterial proteases towards collagen; (**b**) fresh water bacterial proteases towards collagen; (**c**) marine bacterial proteases towards muscle protein; and (**d**) fresh water bacterial proteases towards muscle protein. Values were displayed as means ± SD (*n* = 3).

The collagen and muscle proteins from salmon byproducts were both hydrolyzed with different kinds of bacterial proteases, respectively. The hydrolysis results of 5 min were analyzed by sodium dodecyl sulfate polyacrylamide gel electrophoresis (SDS-PAGE) (Figure 3) and the rate of hydrolysis of each bacterial crude enzyme was also measured (Figure 4). The antioxidant activities of each hydrolysis product after 30 min were measured. As depicted in Figure 3, collagen obtained from salmon skin through hot water treatment consisted of proteins of similar molecular weight. The triple α-helical structure of marine-sourced collagen was unstable at high temperature; it could convert from tight form to relaxed form, and part of the collagen fiber would be broken during hot water treatment. That might be the reason why the salmon collagen extracted in hot water did not display as several specific bands. It could be observed that crude enzymes from *Pseudoalteromonas* sp. J2, *Pseudoalteromonas* sp. SQN1, *Vibrio* sp. SQS2-3, *Photobacterium* sp. YJ2, *Bacillus* sp. TC3, *Bacillus* sp. MH12, *Aeromonas* sp. ZM3, and *Aeromonas* sp. ZM7 could hydrolyze collagen into smaller pieces more effectively. Compared with the results of substrate-immersing zymography, those crude enzymes that degraded collagen quickly commonly contained several proteases or formed brilliant bands in gel, which indicated high enzymatic activity. The antioxidant activity of each group was measured through DPPH, hydroxyl radical scavenging assays, and ferrous ion chelating assay (Figure 3). The products with a higher hydrolysis degree tend to display strong activity in DPPH and hydroxyl radical scavenging assays, such as J2-C (47.77% ± 1.78% in DPPH and 32.75% ± 3.49 in ^●^OH), SQS2-3-C (44.08% ± 1.77% in DPPH and 25.67% ± 3.38% in ^●^OH), and ZM3-C (42.36% ± 1.61% in DPPH and 44.88% ± 1.70% in ^●^OH). These results indicated that those antioxidant peptides scavenging free radicals directly were closely related to the degree of hydrolysis or molecular weight. Peptides with small molecular weight could react with free radicals more easily and displayed stronger antioxidant activity [17]. However, collagen hydrolysates with higher hydrolysis degree did not show significant activity in Fe^2+^ chelating assay. Contrarily, the MH2-C and ZM8-C groups with lower hydrolysis degree displayed relatively stronger Fe^2+^ chelating activity (21.75% ± 2.87% and 20.96% ± 2.44%).

Previous studies commonly hydrolyzed collagen with commercial proteases. For example, Wang et al. used trypsin and pepsin to degrade collagen from a croceine croaker for 4 h, and obtained products with hydroxyl radical scavenging activity (53.11% ± 0.97% and 44.96% ± 1.97%, at a concentration of 10 mg/mL, respectively) [18]. Yang et al. reported that bromelain or papain could finish the digestion of retorted gelatin from cobia skin within 0.5 h, producing antioxidant fractions, while pancreatin or trypsin needed at least 2 h [19]. Mendis et al. obtained antioxidant peptides from the skin gelatin of jumbo squid with trypsin for 4 h [20]. Compared with the crude enzymes used in this study, most of the commercial proteases took longer to produce antioxidant peptides. It was common that commercial proteases were single-enzyme, while crude enzymes obtained from bacterial fermentation were multi-enzymes. Multi-proteases possessed more cleavage sites. More potential bioactive peptides would be released and the reaction time could be shortened.

The protein component from salmon muscle was more complicated than collagen, which contained a series of proteins with different molecular weights (Figure 3). In addition, it was much more difficult for enzymes to hydrolyze muscle proteins compared with collagen. In the first five minutes, *Bacillus* sp. TC3 and *Bacillus* sp. MH12 could degrade most of the protein from salmon muscle. *Pseudoalteromonas* sp. SQN1, *Vibrio* sp. SQS2-3, *Photobacterium* sp. YJ2, *Bacillus* sp. MH12, *Aeromonas* sp. ZM3, and *Aeromonas* sp. ZM7 could hydrolyze part of muscle protein. In DPPH and hydroxyl radical scavenging assays, the antioxidant activity of muscle hydrolysate was much stronger than that of collagen hydrolysate. The product of muscle hydrolyzed with enzyme from *Pseudoalteromonas* sp. SQN1 showed the strongest activity after 30 min hydrolysis (74.06% ± 1.14% in DPPH and 69.71% ± 1.97% in ^●^OH). Compared with the Fe^2+^ chelating assay results of collagen hydrolysates, muscle protein hydrolysates generally displayed stronger activity, especially the MH2-M and ZM-8 groups, which exhibited significant activity (55.52% ± 4.51% and 41.42% ± 2.29%, respectively). Similar to collagen hydrolysates, those muscle protein hydrolysates with a lower hydrolysis degree displayed higher Fe^2+^ chelating activity. It was possible that a suitable hydrolysis degree was an important factor in preparing peptides with better ion chelating activity. It was also reported that the amino acid residues of source protein could affect the antioxidant activity of peptides [17]. Nazeer et al. used gastrointestinal digestive enzymes to hydrolyze croaker (*Otolithes ruber*) muscle proteins and prepared a peptide Lys-Thr-Phe-Cys-Gly-Arg-His with strong DPPH and hydroxyl radical scavenging activity (84.5% ± 1.2% and 62.4% ± 2.9%) [21]. Chi et al. used trypsin to hydrolyze monkfish muscle proteins and prepared three peptides Glu-Trp-Pro-Ala-Gln, Phe-Leu-His-Arg-Pro, and Leu-Met-Gly-Gln-Trp. All of these peptides displayed strong activities in DPPH (EC_50_ 2.408, 3.751, and 1.399 mg/mL), hydroxyl radical (EC_50_ 0.269, 0.114, and 0.040 mg/mL), and superoxide anion radical (EC_50_ 0.624, 0.101, and 0.042 mg/mL) scavenging assays [20]. Most of these reported antioxidant peptides contained specific amino acid, such as cysteine, tyrosine, histidine, and so on, and they made a great contribution to remove free radical or chelate oxidation-related ions [17]. The Gly-X-Y repeating sequence made the amino acid composition of collagen simple and rich in glycine and proline, which do not possess strong active sites against free radicals. The amino acid composition of muscle proteins was more complicated than that of collagen, which may exist more potential antioxidant peptide sequences. In addition, the muscle proteins were much more stable than collagen. Extracted with homogenization, the structure of the muscle proteins could be kept intact, which ensures that the activity of the products was very similar even though they were prepared in different batches. Therefore the muscle was more suitable than collagen to be used in antioxidant peptide preparation.

### 2.3. Optimization of Hydrolysis Condition

**Figure 5:**
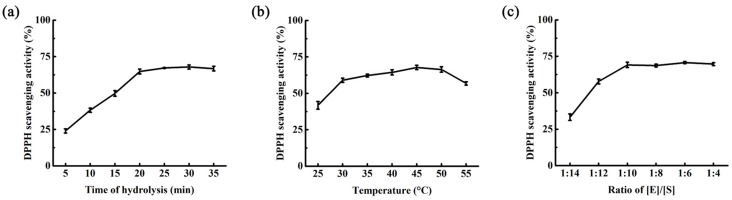
Single-factor analysis of muscle protein hydrolysis with SQN1: (**a**) incubated at 50 °C with a ratio of [E]/[S] in 1:10 for 5, 10, 15, 20, 25, 30, and 35 min; (**b**) incubated at 25, 30, 35, 40, 45, 50, and 55 °C with a ratio of [E]/[S] in 1:10 for 30 min; (**c**) incubated at 50 °C for 30 min with a ratio of [E]/[S] in 1:4, 1:6, 1:8, 1:10, 1:12, and 1:14. Values were displayed as means ± SD (*n* = 3).

A single factor analysis towards the hydrolysis of muscle protein with enzyme from *Pseudoalteromonas* sp. SQN1 was carried out, including time of hydrolysis, temperature of hydrolysis, and the ratio of [E]/[S] (Figure 5). Then the time, temperature, and ratio of [E]/[S] were selected to be 25 min, 45 °C, and 1:50 (g/g). The central composite design was designed with Design Expert 8.0. The experimental conditions and DPPH scavenging activity are listed in Table 2. Variance analysis of linear model with ANOVA is displayed in Table 3, which indicates that the ratio of [E]/[S] was a significant factor influencing the DPPH scavenging activity of muscle protein hydrolysate.

### 2.4. Purification of Antioxidant Peptides from Hydrolysate of Muscle Proteins

**Figure 6:**
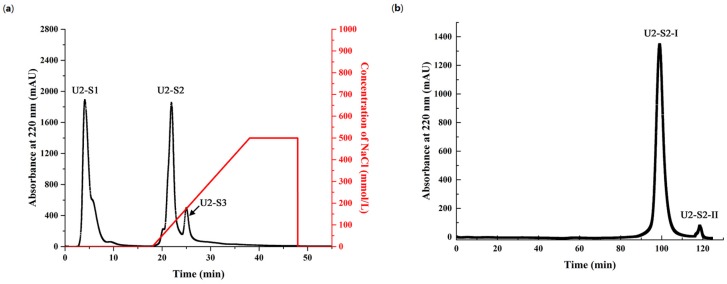
Purification of antioxidant fractions with fast protein liquid chromatography on (**a**) Macro-Prep High S column and (**b**) Sephadex G-15 column.

Since the crude enzyme from *Pseudoalteromonas* sp. SQN1 could hydrolyze salmon muscle proteins to release peptides with strong antioxidant activity, the active fractions were further purified with ultrafiltration, cation exchange chromatography, and size exclusion chromatography gradually. Ultrafiltration tubes with 3 kDa molecular weight cutoff (MWCO) were selected to separate small peptides after 30 min hydrolysis. As shown in Table 4, the DPPH and hydroxyl radical scavenging activity of muscle hydrolysis product (IC_50_ 0.721 ± 0.024 mg/mL and 1.371 ± 0.178 mg/mL) was higher than the activity of smaller fraction U2 (IC_50_ 0.377 ± 0.013 mg/mL and 0.882 ± 0.127 mg/mL) but lower than U1, which was the fraction with a larger size (IC_50_ 0.972 ± 0.031 mg/mL and 1.495 ± 0.214 mg/mL). This indicated that peptides with smaller size were the major active fraction in this product, and therefore U2 was chosen to be further purified with cation exchange chromatography. As shown in Figure 6a, U2 was separated into three major fraction peaks, which were collected, lyophilized, and detected. The first eluted peak (U2-S2) showed the strongest antioxidant activity (IC_50_ 0.289 ± 0.022 mg/mL and 0.681 ± 0.078 in DPPH and ^●^OH). This fraction was further purified with a Sephadex G-15 size exclusion column (Uppsala, Sweden), and two fractions (U2-S2-I and U2-S2-II) were obtained (Figure 6b). U2-S2-I accounted for 99.03% of U2-S2 according to the integral area calculation of Bio-Rad ChromLab software (Hercules, CA, USA). Furthermore, this fraction also displayed similar DPPH and hydroxyl radical scavenging activity (IC_50_ 0.263 ± 0.018 mg/mL and 0.512 ± 0.055 mg/mL) compared with U2-S2. This result showed that U2-S2-I was the major active fraction. As shown in Table 5, the fraction U2-S2-I displayed higher DPPH scavenging activity compared with other reported antioxidant peptides purified from different muscle protein hydrolysate. This indicated that salmon muscle hydrolyzed by protease from *Pseualtermonas* sp. SQN1 would be a feasible method to prepare antioxidant peptides.

### 2.5. Oxygen Radical Absorption Capability (ORAC) Assay

**Figure 7:**
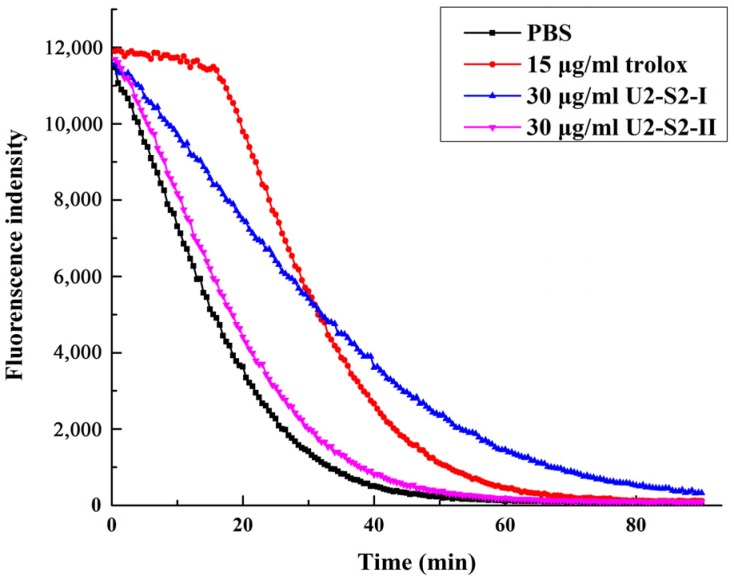
Oxygen radical absorption capability of U2-S2-I and U2-S2-II compared with PBS control group and trolox-positive group.

Oxygen radical absorption capability assay was used to detect the antioxidant activity against peroxyl radicals [27]. Peroxyl radical was considered to be the major free radical generated during the auto-oxidation process of lipid and fatty acid. Peptides with strong absorption capability against peroxyl radical could be a potential antioxidant additive in the food processing industry. The activities of two fractions separated in size exclusion chromatography were detected with this method. The decay speed of the fluorescence curve reflected the reaction speed of the peptides and the area under the curve reflected the quantity of the peroxyl radical removed by antioxidant. As shown in Figure 7, U2-S2-I displayed its effect in decreasing the decay of fluorescence and its antioxidant activity (1.960 ± 0.381 mmol·TE/g) was much stronger than U2-S2-II (0.344 ± 0.079 mmol·TE/g). Antioxidant-donating hydrogen could block the radical chain reaction caused by peroxyl radical in ORAC assay. The result indicated that U2-S2-I might contain active peptides working as hydrogen donors. Specific amino acid residues, such as cysteine, tyrosine, and histidine, could provide hydrogen for free radicals from the sulfydryl group (-SH), the phenolic hydroxyl group, and the iminazole circle, respectively. Meanwhile, specific amino acid residues could also form a stable structure to stop the radical chain reaction.

### 2.6. DNA Protection Effect against Oxidation-Induced Damage

**Figure 8:**
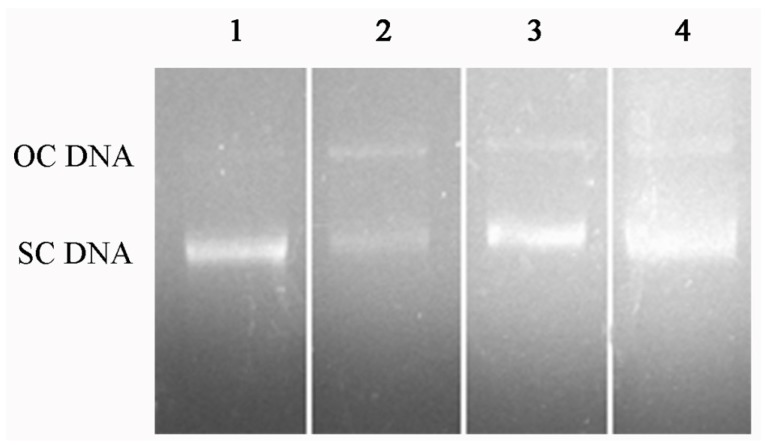
DNA protection effect of U2-S2-I against oxidation-induced damage. Lane 1: plasmid DNA pET-22b without oxidation damage; Lane 2: plasmid DNA pET-22b without antioxidant was attacked by hydroxyl radical; Lane 3: plasmid DNA pET-22b was protected by U2-S2-I against the attack of hydroxyl radical; Lane 4: plasmid DNA pET-22b was protected by SQN1-M hydrolysate against the attack of hydroxyl radical.

Hydroxyl radicals are known for causing oxidative breaks in DNA strands. The DNA protection effect of U2-S2-I was examined using plasmid DNA in vitro compared with the initial hydrolysate. The results in Figure 8 showed that the concentration of supercoil DNA significantly decreased, and the open circle DNA appeared in damage group. When the plasmid DNA was exposed to U2-S2-I or initial hydrolysate, the concentration of supercoil DNA was still high. This indicated that both the hydrolysis product and the final purified fractions have an antioxidant effect. In addition, the antioxidant effect of these two groups was similar, which also indicates that the final fraction U2-S2-I might be the major active fraction of this hydrolysis product. DNA damage is a typical phenomenon of cytopathy caused by oxidative stress in vivo. Peptides with a DNA protection effect against oxidation could be further developed as a functional supplement to prevent diseases related to oxidation. Sheih et al. prepared an antioxidant peptide with DNA protection effect from algae protein hydrolysate, and the peptide could increase the viability of AGS cells [28]. Karawita et al. found that the enzymatic extracts of microalgae could effectively inhibit DNA damage and repair H_2_O_2_-induced DNA damage in mouse lymphoma L5178 cells [29].

## 3. Experimental Section

### 3.1. Materials

Fresh salmon skin with muscle was purchased from a seafood market in Shanghai, China, and was stored at −20 °C prior to use. The soybean meal, corn powder, and wheat bran were purchased from a supermarket in Changsha, Hunan province, China. Tryptone and yeast extraction were purchased from Thermo Fisher Oxoid (Basingstoke, Hamshire, UK). Sephadex G-15 size exclusion gel was purchased from GE Healthcare Life Sciences (Uppsala, Sweden). The other regents used are commercially available.

*Pseudoalteromonas* sp. J2, *Pseudoalteromonas* sp. SQN1, *Bacillus* sp. SQN5, *Vibrio* sp. SQS2-3, *Vibrio* sp. SWN2, and *Photobacterium* sp. YJ2 were from the inshore environment of the South China Sea. *Bacillus* sp. TC3, *Exiguobacterium* sp. MH2, *Bacillus* sp. MH12, *Aeromonas* sp. ZM3, *Aeromonas* sp. ZM7, *Paenibacillus* sp. ZM8, and *Pseudomonas* sp. ZM9 were from the lakes on the Yungui plateau.

### 3.2. Preparation of Bacterial Extracellular Proteases

#### 3.2.1. Preparation of Bacterial Proteases from Fermentation

The method of proteases preparation was modified according to Liu’s study [30]. The protease-producing bacteria were activated in a 2216E medium with shaking at 200 rpm and 18 °C. When the OD_600_ value reached 0.6, the bacteria were incubated in a fermentation broth (0.5% corn powder, 0.5% bean powder, 0.25% wheat bran, 0.1% CaCl_2_, 0.4% Na_2_HPO_4_, and 0.03% KH_2_PO_4_, prepared with sea water) [31] and fermented at 200 rpm and 18 °C for 5fivedays. The supernatant was centrifuged at 12,000× *g* and 4 °C for 30 min to collect the crude proteases.

#### 3.2.2. Detection of Proteases with Substrate-Immersing Zymography

Substrate immersing zymography was modified to detect the proteolytic activities according to the method developed by Liu [30]. Crude proteases (16 μL) were loaded in a 12.5% SDS-PAGE gel with constant voltage at 100 V for 10 min and 160 V for 50 min in proper order. The gel was washed three times with 2.5% Triton X-100 for 15 min to remove SDS after electrophoresis. Then the gel was washed with 50 mM Tris-HCl (pH 8.0) and immersed in 0.1% casein at 37 °C for 60 min. Subsequently, the gel was stained with 0.1% Coomassie Brilliant Blue R-250 for 3 h, and destained by ethanol/acetic acid/H_2_O (2:1:7) mixture with shaking until the bands of proteolytic activity became visible.

### 3.3. Protein Hydrolysis of Salmon Byproducts Using Bacterial Extracellular Proteases

The salmon byproducts consisted of fish skin and subcutaneous muscle, which were separated and pretreated in different ways. The total protein concentration of crude enzymes from different bacteria was diluted to 1 mg/mL.

#### 3.3.1. Hydrolysis of Salmon Collagen Using Bacterial Extracellular Proteases

Fish skin was cut into pieces and washed with cold distilled water three times. Then 5 g fish skin was cooked in 50 mL distilled water at 75 °C for 30 min. The supernatant was collected by centrifugation at 12,000× *g* for 15 min. Salmon skin collagen was lyophilized for 24 h until the protein changed into a solid. Then the collagen was dissolved into ddH_2_O to the concentration of 10 mg/mL, then mixed with different kinds of bacterial extracellular proteases at an enzyme/substrate ratio of 1:90 (*g*/*g*), and the mixtures were incubated at 50 °C. After 5 min, 20 μL of hydrolysates were sampled and inactivated at 90 °C for 10 min. The hydrolysis of collagen was detected by 12.5% SDS-PAGE. The hydrolysis rates of collagen with different bacterial crude enzyme were quantified as follows:

Rate of hydrolysis (μmol/min·g) = (*c*_t_ − *c*_0_) × 0.18 mL/(5 min × 1 mg/mL × 0.02 mL),

where *c*_0_ and *c*_t_ were defined as the concentration of peptides product before and after hydrolysis, respectively. After 25 min, the mixtures of hydrolysis were inactivated at 90 °C for 10 min.

#### 3.3.2. Hydrolysis of Salmon Muscle Using Bacterial Extracellular Proteases

The salmon muscle was homogenized at a speed of 10,000 rpm for 2 min in distilled water. Then the supernatant was collected by centrifugation at 13,000× *g* for 30 min. The concentration of muscle proteins was quantified and diluted to 5 mg/mL with BCA method. Then the muscle protein was mixed with different proteases at an enzyme/substrate ratio of 1:45 (*g*/*g*), and the mixtures were incubated at 50 °C. After 5 min, 20 μL of hydrolysates were sampled and inactivated at 90 °C for 10 min. The hydrolysis of collagen was detected by 12.5% SDS-PAGE. The hydrolysis rates of muscle protein with different bacterial crude enzyme were quantified using the method described in Section 3.3.1. After 25 min, the mixtures of hydrolysis were inactivated at 90 °C for 10 min.

### 3.4. Isolation of Antioxidant Peptides from Muscle Hydrolysate

#### 3.4.1. Isolation of Muscle Hydrolysate by Ultrafiltration

After being hydrolyzed by proteases from *Pseudoalteromonas* sp. SQN1, the hydrolysate of salmon muscle proteins was centrifuged in ultrafiltration tube with 3 kDa molecular weight cutoff (Millipore, Temecula, CA, USA) at 5000× *g* for 30 min to isolate fractions above 3 kDa (MUF-1) and below 3 kDa (MUF-2). The antioxidant activities of these two fractions were detected by hydroxyl radical scavenging assay.

#### 3.4.2. Cation Exchange Chromatography

The active fraction MUF-2 was further purified in a Bio-Rad Macro-Prep High S column (5 × 1.26 cm) with NGC chromatography system (Bio-Rad, Hercules, CA, USA). The column was equilibrated with distilled water. Then 5 mL MUF-2 were loaded into the pre-equilibrated column at a flow rate of 1.5 mL/min, and eluted with distilled water for 18 min at a flow rate of 1.5 mL/min. Then the column was eluted with a linear gradient of 1 M NaCl (0%–50%) at a flow rate of 1.5 mL/min for 20 min. Peptide fractions were monitored at 220 nm and collected at a volume of 5 mL. All the fractions were lyophilized.

#### 3.4.3. Size Exclusion Chromatography

After cation exchange chromatography, the fraction with the strongest activity was further purified by Sephadex G-15 size exclusion column (1.6 × 80 cm) with NGC chromatography system (Bio-Rad). Fraction was dissolved in 1 mL distilled water and loaded into a column pre-equilibrated with distilled water. The column was eluted with distilled water at a flow rate of 0.75 mL/min. Peptide fractions were monitored at 220 nm and collected at a volume of 5 mL. All the fractions were lyophilized and the antioxidant activities were evaluated by DPPH radical scavenging assay.

### 3.5. Evaluation of Antioxidant Activity

#### 3.5.1. DPPH Radical Scavenging Activity

The DPPH radicals scavenging activity assay was measured according to the method of Shimada et al. [32]. One hundred microliters of DPPH solution (0.1 mM in 95% ethanol) were mixed with 20 μL of purified fraction solution in an Eppendorf tube to initiate the reaction, which was incubated at room temperature for 60 min. Then the reaction mixture was transferred into a 96-well microplate. The absorbance of the resulting solution was measured at 517 nm using an Enspire 2300 microplate reader (Perkin Elmer, Waltham, MA, USA). For the blank, the purified fraction was replaced with distilled water. The DPPH radicals scavenging activity was calculated using the following formula:

DPPH radical scavenging activity (%) = [1 − *ABS*_sample_/*ABS*_blank_] × 100%.

#### 3.5.2. Hydroxyl Radical Scavenging Activity

Hydroxyl radical scavenging activity was measured according to the method developed by Wang et al. [33]. Hydroxyl radicals were generated by the Fenton reaction. Ferrous ion (Fe^2+^) could combine with 1,10-phenanthroline to form red compounds with a maximum absorbance at 536 nm. The absorbance value would decrease when ferrous ion was oxidized into ferric ions by a hydroxyl radical, which reflected the concentration of hydroxyl radicals. In this system, 1,10-phenanthroline (40 μL, 2 mM) and the sample (80 μL) were added into an Eppendorf tube and mixed. The FeSO_4_ solution (40 μL, 2 mM) was then pipetted into the mixture. The reaction was initiated by adding 40 μL H_2_O_2_ (0.03% *v*/*v*). After being incubated at 37 °C for 60 min, the reaction mixture was transferred into a 96-well microplate. The absorbance of the resulting solution was measured at 536 nm using an Enspire 2300 Multimode Plate Reader (Perkin Elmer). The group without any antioxidant was used as a negative control, while the mixture without H_2_O_2_ was used as the blank. The hydroxyl radical scavenging activity (HRSA) was calculated as follows:

HRSA (%) = [(*A*_s_ − *A*_n_)/(*A*_b_ − *A*_n_)] × 100%,

where *A*_s_, *A*_n_, and *A*_b_ were the absorbance values of the sample, the negative control, and the blank determined at 536 nm after the reaction, respectively.

#### 3.5.3. Ferrous Ion Chelating Assay

The ferrous ion chelating activity was measured according to the method of Thiansilakul [34]. Fresh ferrous sulfate (2 mM, 40 μL) and a 400-μL sample were mixed together, and the ferrous ion was detected by 80 μL 5 mM ferrozine. Distilled water was added into the mixture until the total volume reached 2 mL. Then the mixture was incubated at 37 °C for 20 min. The absorbance of the reaction product was measured at 562 nm, and the chelating ratio was calculated as follows:

Fe^2+^ chelating ratio (%) = 1 − (*A*_s_/*A*_c_) × 100%,

where *A*_s_ and *A*_c_ were the absorbance values of the sample group and the control group determined at 562 nm, respectively.

#### 3.5.4. Oxygen Radical Absorbance Capability (ORAC) Assay

The ORAC assay was measured according to the method developed by Alberto et al. [35]. Sample solution (20 μL) and fluorescein (100 μL, 96 nM) were added into a 96-well microplate and pre-incubated at 37 °C in Enspire 2300 Multimode Plate Reader (Perkin Elmer). The reaction was initiated by adding 30 μL pre-warmed AAPH (320 mM). The reaction was performed at 37 °C. The fluorescence intensity was measured every 30 s for 180 cycles with excitation and emission wavelengths of 485 nm and 538 nm, respectively. Trolox was used as a positive control. The ORAC was defined as the trolox equivalent (mmol·TE/g) according to the area under the curve (AUC), and was calculated as follows:

ORAC = (*AUC*_sample_ − *AUC*_control_)/(*AUC*_Trolox_ − *AUC*_control_) × (*M*_Trolox_/*M*_sample_),

where *AUC*_sample_, *AUC*_control_, and *AUC*_Trolox_ were the integral areas under the fluorescence decay curve of the peptide, 75 mM PBS (pH 7.4), and Trolox, respectively. *M*_Trolox_ and *M*_sample_ are the concentrations of trolox and peptide.

#### 3.5.5. Protection Effect on Oxidation-Induced DNA Damage

The protection effect on oxidation-induced DNA damage assay was modified according to the method described by Qian et al. [36]. Plasmid DNA displays different structures and a different running rate in agarose gel electrophoresis according to the damage degree. The reaction system included 6 μL of pET-22b DNA, 3 μL of 2 mM FeSO_4_, 6 μL of antioxidant, and 5 μL of 0.3% H_2_O_2_. The mixture was incubated at 37 °C for 10 min, and then analyzed by 1% agarose gel electrophoresis at a constant voltage (130 V) for 30 min. The gel was then monitored in a gel imaging system (Bio-Rad) after being immersed in ethidium bromide for 15 min.

### 3.6. Statistical Analysis

All experiments were conducted in triplicate (*n* = 3). The values were expressed as mean ± standard deviation, which were calculated with the Origin 9.1 software. An ANOVA test was used to analyze data in the SPSS 19.0 software.

## 4. Conclusions

Marine protein resources are considered to be a huge treasury of bioactive peptides. More and more researchers are attempting to prepare novel bioactive peptides from marine protein through enzymatic hydrolysis. This study proved that muscle proteins from salmon byproducts were more suitable to be used as a preparation material of antioxidant peptides compared with collagen from salmon byproducts. Moreover, the antioxidant peptide fraction exhibited a DNA protection effect, which could be developed as a potential dietary supplement to prevent oxidation-related diseases. In addition, commercial proteases were the first choice to be used in protein hydrolysis in previous studies, because these proteases are relatively thoroughly studied and are easily obtained from supermarkets. However, non-commercial proteases from bacteria also possess great potential in bioactive peptide preparation, due to their high efficiency and low cost.

## Figures and Tables

**Table 1:** Summary of crude enzymes from bacterial fermentation.

Bacteria Strain	Concentration of Total Protein (mg/mL)	Specific Activity (U/mg)	Amount of Proteases	Molecular Weight (kD)
Bacteria from sea water				
*Pseudoalteromonas* sp. J2	2.63	39.06	3	90, 40, 30~40
*Pseudoalteromonas* sp. SQN1	1.26	347.27	2	90~120, 40
*Bacillus* sp. SQN5,	1.98	161.06		
*Vibrio* sp. SQS2-3	1.18	293.95	3	60, 40, 30
*Vibrio* sp. SWN2	1.65	236.92		
*Photobacterium* sp. YJ2	2.00	54.93	1	14
Bacteria from fresh water				
*Bacillus* sp. TC3	1.03	110.04	1	<14
*Exiguobacterium* sp. MH2	2.20	103.67		
*Bacillus* sp. MH12	3.84	208.78	2	<14
*Aeromonas* sp. ZM3	5.35	99.47	Proteases cannot be separated	
*Aeromonas* sp. ZM7	5.26	19.25	Proteases cannot be separated	
*Paenibacillus* sp. ZM8	1.02	114.68		
*Pseudomonas* sp. ZM9	1.06	168.12	1	30~40

**Table 2:** Optimization of muscle protein hydrolysis condition through central composite design (CCD).

Group	Time (min)	Temperature (°C)	Ratio of [E]/[S] (g/g)	DPPH Scavenging Activity (%)
1	29.2	45	1:50	68.13
2	25	45	1:50	68.70
3	25	45	1:50	69.27
4	22.5	47.5	1.5:50	71.19
5	20.8	45	1:50	72.91
6	25	45	1:50	70.80
7	25	40.8	1:50	69.46
8	27.5	42.5	0.5:50	70.99
9	25	45	1.8:50	74.44
10	27.5	42.5	1.5:50	76.54
11	25	49.2	1:50	69.66
12	27.5	47.5	0.5:50	67.93
13	25	45	1:50	73.67
14	25	45	1:50	71.95
15	25	45	1:50	73.48
16	22.5	42.5	1.5:50	73.29
17	22.5	47.5	0.5:50	69.08
18	22.5	42.5	0.5:50	70.42
19	27.5	47.5	1.5:50	70.80
20	25	45	0.2:50	70.43

**Table 3:** Variance analysis of linear model with ANOVA.

Source	Sum of Square	df	Mean Square	*F* Value	*p*-Value (Prob > *F*)
Model	0.390 × 10^−3^	3	1.230 × 10^−3^	6.53	0.0043
A-Time	9.880 × 10^−5^	1	9.880 × 10^−5^	0.52	0.4793
B-Temperature	3.781 × 10^−4^	1	3.781 × 10^−4^	2.01	0.1756
C-Ratio of [E]/[S]	3.213 × 10^−3^	1	3.213 × 10^−3^	17.07	0.0008
Residual	3.013 × 10^−3^	16	1.883 × 10^−4^		
Lack of Fit	2.197 × 10^−3^	11	1.998 × 10^−4^	1.23	0.4375
Pure Error	8.152 × 10^−4^	5	1.630 × 10^−4^		
Cor Total	6.703 × 10^−3^	19			

**Table 4:** DPPH scavenging activity of purified fractions in each step.

Preparation Step	Fractions	IC_50_ Value (mg/mL)		Yield (%)
		DPPH	^●^OH	
Enzymatic hydrolysis	Hydrolysate	0.721 ± 0.024	1.371 ± 0.178	100
Ultrafiltration	U1	0.972 ± 0.151	1.495 ± 0.214	35.18
	U2	0.377 ± 0.013	0.882 ± 0.127	
Cation exchange chromatography	U2-S1	1.781 ± 0.048	1.689 ± 0.118	12.81
	U2-S2	0.289 ± 0.022	0.681 ± 0.078	
	U2-S3	0.972 ± 0.053	0.920 ± 0.093	
Size exclusion chromatography	U2-S2-I	0.263 ± 0.018	0.512 ± 0.055	12.68
	U2-S2-II	4.832 ± 0.552	3.191 ± 0.323	

**Table 5:** Comparing with reported antioxidant peptides sourced from muscle protein.

Source	Enzyme	Antioxidant Activity (DPPH)	Reference
Salmon muscle	Protease from *Pseudoalteromonas* sp. SQN1	IC_50_ = 0.51 mg/mL	
*Scorpaena notata* muscle	neutral serine protease	IC_50_ = 0.60 mg/mL	[22]
Croceine croaker muscle	pepsin and alcalase	IC_50_ = 1.35 mg/mL	[23]
Monkfish muscle	trypsin	IC_50_ = 1.40 mg/mL	[24]
Smooth hound muscle	gastrointestinal proteases	IC_50_ = 0.60 mg/mL	[25]
*Sphyrna ewini* muscle	papain	IC_50_ = 3.06 mg/mL	[26]
